# “Incidentaloma” of the Liver: Management of a Diagnostic and Therapeutic Dilemma

**DOI:** 10.1155/2012/891787

**Published:** 2012-08-08

**Authors:** Denis Ehrl, Katharina Rothaug, Peter Herzog, Bernhard Hofer, Horst-Günter Rau

**Affiliations:** ^1^Department of Visceral, Thoracic und Vascular Surgery, Clinic of Dachau, 85221 Dachau, Germany; ^2^Department of Radiology, Clinic of Dachau, Krankenhausstrare 15, 85221 Dachau, Germany

## Abstract

The continuous development of highly sensitive clinical imaging increased the detection of focal lesions of the liver. These accidentally detected liver tumors without liver-specific symptoms such as cholestasis have been named “incidentalomas.” Diagnostic tools such as sonography, computed tomography, or magnetic resonance imaging are used increasingly in asymptomatic individuals without defined suspected diagnoses in the setting of general prevention or followup after a history of malignancy. But despite continuous improvement of diagnostics, some doubt regarding the benign or malign behavior of a tumor remains. In case an asymptomatic hemangioma or FNH can be preoperatively detected with certainty, the indication for surgery must be very strict. In case of symptomatic liver lesions surgical resection should only be indicated with tumor-specific symptoms. In the remaining cases of benign lesions of the liver, a “watch and wait” strategy is recommended. In case of uncertain diagnosis, especially in patients with positive history of a malignant tumor or the suspected diagnosis of hepatocellular adenoma, surgical resection is indicated. Due to the continuous improvement of surgical techniques, liver resection should be done in the laparoscopic technique. Laparoscopic surgery has lower morbidity and shorter hospitalization than open technique.

## 1. Introduction

In recent years the rapid development of highly sensitive clinical imaging has led to the detection of focal lesions of the liver more frequently. In addition, diagnostic tools are used increasingly in asymptomatic individuals without defined suspected diagnoses in the setting of general prevention or followup after a history of malignancy [1–4]. Unfortunately, the histological nature of a hepatic tumor is rarely proven by one method of imaging, and even sophisticated technologies some doubt regarding the benign or malign behavior of a tumor remain in 10–40% [5, 6]. These accidentally detected liver tumors without liver-specific symptoms such as cholestasis or portal hypertension have been named “incidentalomas” [4]; the reported incidence of these findings ranges from 10.2 to 52% [7, 8]. Autopsy studies have demonstrated up to 52% benign liver lesions in the western population [9, 10]. Other authors could demonstrate an incidence of incidentalomas of 10.2–14.3% of CT scans [7–9]. Generally, these tumors can be true benign or malign neoplasms or so-called tumor-like lesions [3]. Malignant tumors of the liver become usually only in stages of an advanced disease symptomatic. In case of metastases usually a primary tumor is known from the patient's history or can be diagnosed by endoscopy and thoracical and abdominal CT scans [2, 3]. 

Currently, there are no evidence-based guidelines regarding the appropriate approach to diagnosis, interpretation of imaging and laboratory findings, and the indication for surgical resection. Prospective, sufficiently powered, randomized controlled trials on the elective resection of benign liver lesions are lacking. Most recommendations are based on retrospective data with fewer than 60 patients or casuistic reports [1, 10, 11]. We want to add our personal experience and results to the upcoming discussion. 

Generally a primarily conservative approach is considered to be the method of first choice in the treatment of proven benign liver tumors [1, 3, 5, 10, 11]. In total for about 5% of newly diagnosed benign liver tumors, surgery is warranted [10]. Despite the continuous improvement of the radiological and nuclear medical diagnostics, surgery is often just indicated because of the possibility of a primary or secondary malignant tumor in the liver [12–15]. Other common indications for surgical resection include abdominal discomfort, tumor growth, history of adenoma, and rarely the desperate request of the patient [10, 11, 13]. An accepted emergency indication is acute bleeding of a benign liver lesion [16, 17]. This may occur as a free rupture with haemoperitoneum, as prolonged intrahepatic haemorrhage or hemobilia leading to gastrointestinal bleeding, the latter being considerably difficult to diagnose [16, 17]. In spite of the low morbidity and minimal mortality of liver resections in specialized institutions, surgical treatment of benign liver lesion is only justified in the above indications and requires precise criterions [3–5].

The cavernous and capillary hepatic hemangiomas, the focal nodular hyperplasia (FNH), and the hepatocellular adenoma are the most common benign lesions of the liver [1, 3, 11] and are presented below.

### 1.1. Hemangioma (Figure 1)

These are usually diagnosed as asymptomatic incidental findings. In addition to nonspecific symptoms, hemangiomas also (rarely) rupture spontaneously or by trauma and then lead to acute hemorrhagic shock with upper abdominal pain [1, 10, 18]. In the worldwide literature a total of only 97 cases with a rupture of a hemangioma have been published, whereas a spontaneous rupture only happened in 47.4% of cases (46) [19]. Further investigation showed that these spontaneously ruptured hemangiomas had a mean size of 11.2 cm [19]. In an acute situation, the immediate restitution of coagulation factors and rarely TAE are methods of choice [1, 10, 11, 18]. TAE teatment of a hemangioma is difficult and due to the aberrant collateral arterial circulation making almost improbable to stop the multiple inflow from different feeding arteries especially through the periphery of the hemangiomas. Despite therapy, in these situations the mortality rate is 30–40% [10].

Hemangiomas rarely occur in association with clinical syndromes. These include most of all the Kasabach-Merritt syndrome and the Blumgart-Bornman-Terblanche syndrome. The Kasabach-Merritt syndrome is characterized by a hemangioma bleeding, thrombocytopenia, and coagulopathy [1, 10]. The Blumgart-Bornman-Terblanche syndrome is accompanied by fever and abdominal pain [1, 20]. These syndromes can evoke minor and major complications and should be treated surgically [1].

Hemangiomas generally have no growth tendency. In the literature, however, cases of hemangioma growth during pregnancy or after estrogen administration are described [1, 10]. Hemangiomas <10 cm should generally not be treated, even before a pregnancy. In case of a planned pregnancy and a size >10 cm, due to the risk of a possible rupture, a definitive treatment should be discussed [1, 10]. Several studies have concluded that a spontaneous rupture of a hemangioma (even while pregnancy) [10, 19] occurs only very rarely, and therefore a prophylactic resection should only be conducted under special conditions and especially with a size of the hemangioma >11 cm [10]. In case of hemangiomas with high growth trend (>3 cm in 12 months), with symptomatic compression symptoms or recurrent pain, which may correlate with hemorrhage into the lesion, surgical intervention should be indicated [18, 19]. Because of hypotension, unexplained anemia, or diagnosis difficulties of the liver lesion, surgical intervention can be rarely necessary. In exceptional cases, individual patient factors, such as an extreme carcinogenicity phobia or high risk of rupture due to a patients career, sometimes require a resection of the lesion [18]. Overall, the indication for surgical intervention should be found cautiously [21, 22].

Treatment of choice is the parenchyma-saving enucleation or the sparing liver resection [10, 11, 18, 21]. In giant centrally located hemangioma a total vascular exclusion of the liver is a useful technical maneuver to save blood while dissecting it. Only in case of an extensive involvement of a liver lobe or central location, an anatomical resection is indicated [11]. In exceptional cases of giant hemangiomas and less functional parenchyma reserve, in the medical literature as a rarity a liver transplant is described [10].

### 1.2. Focal-Nodular Hyperplasia (FNH) (Figure 2)

10–20% of FNHs occur multifocally [1, 3, 10] and in 5–20% of cases these are diagnosed in combination with a hemangioma of the liver [10]. Clinical symptoms and complications are rare [11]. In case of newly diagnosed FNH, setting of oral contraceptives is currently not recommended [10]. The FNH has no malignant degeneration risk [1]. In the medical literature after initial diagnosis of FNH, a “wait and see” strategy is recommended [1, 10, 11]. For followup after 6 to 12 months, an imaging control investigation to identify a possible tendency of growth should be done [10]. Radiological long-term observations are not necessary.

In case of a FNH, there is primarily no indication for surgical intervention [1]. Surgical therapy can be discussed, especially for large expansive growing FNH nodes [5, 10]. This is especially for women wishing to have children clinically relevant. During pregnancy due to hormonal influences, a progressive growth of FNH could occur [10]. In case of an unambiguous preoperative diagnosis and indication for surgical intervention, the atypical resection is the treatment of choice [3, 5, 10, 11]. Often several liver segments are involved, resulting in an extension of the surgical procedure [11]. The literature describes no recurrence after resection [10].

### 1.3. Hepatocellular Adenoma (Figure 3)

This lesion of the liver is often diagnosed as an incidental finding in asymptomatic patients. In association with an adenoma often right-sided upper abdominal pain (80%) with normal liver function values occurs [1, 3, 23, 24]. Rupture and subsequent acute bleeding event of a previously unknown adenoma occur at 10–30% of cases [1, 10, 23]. This spontaneous rupture happens almost exclusively in adenomas >5 cm and is associated with a mortality of 8% [1, 23].

Beside the risk of acute bleeding complications, hepatocellular adenomas have a malignant degeneration risk from 4.2 to 10%, especially in inflammatory adenomas on MRI and/or the possibility of beta-catenin expression [1, 3, 10, 23, 24]. Generally adenomas of liver healthy patients arising by hormonal stimulation should be differentiated from those that arise due to a preexisting disease of the liver (like liver cirrhosis) and can degenerate into a HCC [24, 25]. Risk factors for malignant transformation are size of the adenoma (usually >5 cm), androgen or steroid use, male gender, and glycogen storage disease [24, 25]. 

The adenomatosis, with more than 10 adenomas in an otherwise normal liver, is a special form. This is gender unspecific and has no association with the use of hormones [26, 27].

The diagnostic differentiation from hemangioma is straightforward [3, 11]. The differential diagnosis of FNH is sometimes difficult [11, 24]. Grazioli et al. showed that gadoxetic acid-enhanced MRI (Primovist) facilitates the differentiation of hepatocellular adenoma and FNH [28]. The gadoxetic acid-enhanced MRI distinguishes between adenomas and FNH with sensitivity of 92% and specificity of 91% [28]. The differentiation to (highly) differentiated hepatocellular carcinoma (HCC), especially for the fibrolamellar type (FLC) of HCC is often problematic [29, 30]. For this reason, hepatic resection should be performed according to oncologic criteria of a simple atypical resection up to the extended hemihepatectomy [11, 24, 29, 30]. In nonhemorrhagic, hemorrhagic and especially in inflammatory adenomas on MRI and the possibility of beta catenin expression, the indication for surgery should be clear irrespective of size. Over 45% of adenomas demonstrate a progressive growth, so up to 25% of the operated patients require another surgical intervention [10, 24, 30]. After resection, the mortality is <1% [24, 29, 30]. In order to detect malignant transformation of adenomas in patients after resection, a strict followup is needed, containing annual imaging and regular AFP determination [10]. The liver transplant is considered to be the ultima ratio in case of solid, very large, unresectable, symptomatic adenomas, in distinct adenomatosis of the liver with AFP increase or in case of multiple, progressive growing recurrence adenomas [10, 24].

### 1.4. Tumors of Unknown Dignity

Atypical tumors of the liver, such as the angiolipoma or cystadenoma, often have an inhomogeneous structure that usually a precise preoperative classification of a benign or a malignant tumor is impossible. This is the reason why in case of these tumors oncologic resections with appropriate security clearance are recommended [1, 11].

## 2. Patients and Methods

### 2.1. Tumor Entities

The conservative and surgical treatment of benign lesions of the liver includes several regenerative or real-neoplastic tumor entities. Depending on their origin, these tumors are divided in hepatocellular, endothelial, biliary, mesenchymal, and connective tissue tumors [11] (Table 1).

The benign lesions of the liver can be divided in solid tumors, in tumors with solid areas, or cystic tumors. They are uni- or multilocular [31, 32]. 

As illustrated in Table 2 (for the three most common benign liver tumors), with detailed history and clinical findings, first conclusions regarding the dignity of the tumor can be drawn. In addition tumor-associated demographic data, possible characteristics, and further diagnostic measures are listed in Table 2.

### 2.2. Tumor Diagnostics

Clinical symptoms of the patient are crucial for the extent and type of diagnostic measures to be executed. If the patient is burdened by severe symptoms of the tumor, resection is indicated and further diagnostic workup is dispensable. On the other hand, with an asymptomatic tumor and nonspecific findings, every attempt has to be made to ensure the diagnosis. The contrast medium- (CM-) based computed tomography (CT) (multiphase spiral CT) and magnetic resonance imaging (MRI), especially when using liver-specific CM (e.g., gadoxetic acid (Primovist)), are the methods of choice in the diagnosis of benign liver tumors [33, 34]. 

In clinical routine, sonography is primarily used as screening method. Through the use of CM and technical enhancements, such as tissue harmonic imaging, the importance of sonography in the diagnosis of benign liver lesions has greatly increased. The disadvantage of this method of investigation is the investigator dependency [35, 36]. 

Even nuclear medical procedures, such as the erythrocyte pool scintigraphy with 99Tc or hepatobiliary scintigraphy under the utilization of tumor-specific characteristics without precise morphological mapping of the lesion, can achieve a high sensitivity in diagnostics. Relatively high costs and limited availability preclude nuclear medicine procedures from more extensive use despite their proven diagnostic value [11, 38].  Table 3 summarizes the usual imaging procedures and the respective performance of the three most common benign liver lesions.

In medical literature there are only few publications with larger numbers of cases that match radiological diagnosis with the corresponding histopathologic findings after surgical resection. Grimm et al. showed, in 26 cases of histological confirmed benign liver tumors, that the multiphase CT or MRI examination only in 54% of the cases produced the correct preoperative diagnosis [39]. 

For the highest diagnostic “security” preoperatively, at least one imaging procedure should be performed using a suitable, liver-specific CM in combination with a nuclear medicine examination [11, 12]. As a result of this method, in only about 10% of liver tumors the dignity remains preoperatively unclear [10]. With these diagnostic possibilities, a hemangioma or FNH can be diagnosed with a specificity of <95% and a sensitivity of >80% [1, 11].

In addition to liver enzymes, bilirubin, and cholestasis, the laboratory testing should include the determination of the tumor markers AFP, CA 19-9, and CEA to distinguish it from malignant tumors of the liver [1, 3, 4, 10]. The integrity of liver synthesis can be estimated by serum cholinesterase levels and the quick value. These parameters in combination with the morphological assessment of cirrhosis status and volume rendering of the planned resection allow a reasonable prediction of functional outcome [1, 3, 4].

In case of a symptomatic tumor, determinants of resectability such as size, location, relationship to the hilum, and the blood vessels are in the focus. Thereby a minimum of diagnostics, such as a sectional imaging, is sufficient. If in case of an asymptomatic neoplasia surgery is necessary, a graduated diagnostic procedure should be performed to determine the exact type of tumor and the subsequent appropriate therapy [4, 11].

In the literature, the performance of a percutaneous fine-needle biopsy (FNB) is controversially discussed. Because of the insufficient validity and often missing therapeutic consequences, the FNB should not be performed [12, 40–42]. Only 34–40% of FNB histologies are consistent with the histology of the surgical preparation [4, 10]. The puncture of the liver has a morbidity of 0.5% and a mortality of 0.05% [1, 4, 10]. Another nonnegligible risk is the possible seeding of malignant cells through the puncture of hepatocellular carcinoma (HCC). Huang et al. and Smith showed, through the puncture of the liver in up to 2% of cases, seeding of malignant cells in the needle tract occurs [43, 44]. 

In clinical practice preoperatively, the exact morphology of a tumor in up to 35–45% of cases is not clearly determined by radiological investigations. With the suspected diagnosis of a possible malignancy in these cases, often and completely understandable surgical resection of the lesion is indicated [11].

In case of a suspected adenoma due to the possible risk of malignant transformation or occult malignancy, surgical resection should be done initially or rarely after transarterial embolization (TAE). In acute bleeding with hemorrhagic shock, the extent of the diagnosis depends on the one hand on the urgency of the operation and on the other if the entity of tumor is previously known. In case of a known hemorrhagic hemangioma instead of a risky emergency surgery, a therapeutic TAE could be tried [45, 46]. In case of a nonhemorrhagic adenoma, TAE is not useful. 

### 2.3. Indication

Because of preventive checkups, followup, and screening examinations, increasingly more asymptomatic liver lesions, especially in patients with a positive history of malignant tumor, are newly diagnosed. This has led to a shift in the spectrum of indications for surgery from symptomatic to asymptomatic patients. Depending on the tumor localization, specific symptoms such as a laboratory chemical cholestasis or portal hypertension are possible [1, 3, 10, 11]. Often nonspecific symptoms such as feeling some pain in the right upper abdomen, a feeling of fullness, or a vague dyspnea occur [42, 47]. Only because of a nonspecific pain syndrome, the indication for surgery should be avoided. D'Halluin et al. showed that up to 100% of nonspecific symptoms with conservative therapy are to relieve, especially after finishing oral contraception [48].

The indication for surgery should be primary found in relation to the symptoms and the suspected diagnosis. In descending order in cases of an acute symptomatology such as bleeding, the suspicion of an adenoma or a lesion of unknown dignity, in particular with positive tumor history, a tumor-induced symptomatology, the progression of size, some nonspecific symptoms, or eventually also in case of a cancerophobia of the patient liver resection should be indicated [10–15].

In case of a prior history of hemangioma, if a spontaneous or traumatic rupture happens, the indications are entirely provided for the conservative approach, eventually after successful TAE [11, 18]. TAE treatment of hemangioma is very difficult and for this reason a treatment of second line. 

### 2.4. Surgery

The recommendations for surgical management of benign liver lesions are based on the results of retrospective analysis or case reports with fewer than 60 patients (medical evidence 3-4) [10].

**Figure 4:**
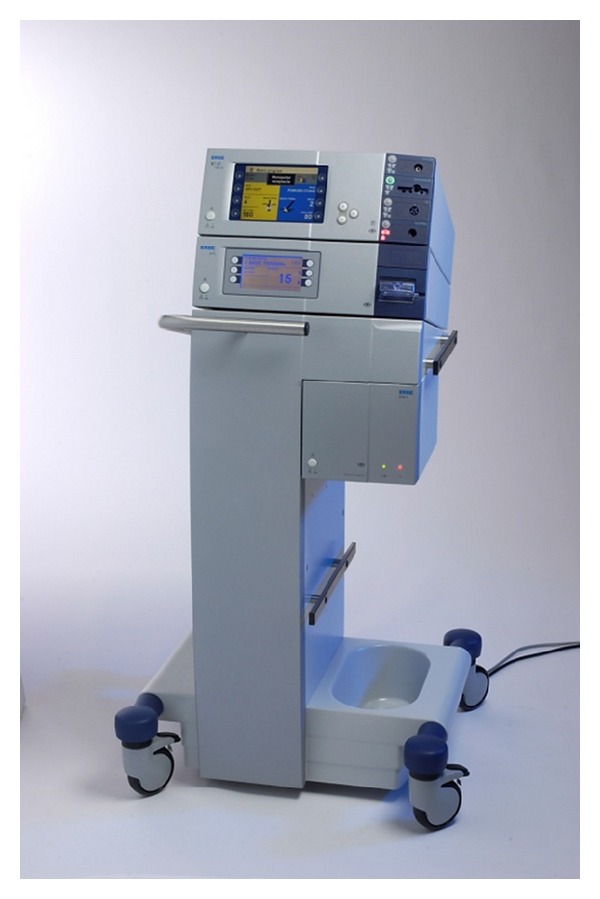
Waterjet dissector (Helix Hydro-Jet; Erbe Elektromedizin GmbH, Tübingen, Germany).

If the indication for surgery was found, parenchyma sparing techniques for operation should be preferred. If the diagnoses of hemangioma or FNH are proven, enucleation is the method of choice. With the enucleation the loss of functional liver parenchyma and, at the same time, blood loss and the risk of bile leaks can be reduced [4, 42, 49]. This leads to a significantly decreased perioperative morbidity and mortality [49]. Peripheral and/or smaller tumors should be resected in minimally invasive laparoscopic technique [10, 50, 51]. In our experience the waterjet dissection offers specific advantages (Figure 4). Compared to resections with the Cavitron ultrasonic surgical aspirator (CUSA) or by blunt dissection of the liver, the intraoperative blood loss and the time for resection can be significantly reduced [4, 51, 52]. In addition, bile ducts can be clearly visualized and closed by a clipping device, thereby avoiding postoperative bilioma. A Pringle maneuver for temporary vascular occlusion is not advocated when using water-jet dissection, because blood loss is minimal even without such measures and reperfusion damage to the residual liver can be significant [52]. The resection surface is meticulously inspected for venous and bile leakage, which can be closed by monofilamentous, resorbable sutures (Monosyn), and the whole plane is sealed by fibrin (TachoSil) [4, 51]. Anesthesiologic management of fluid intake is crucial; to minimize the blood loss at surgery, during resection the lowest possible PEEP and CVP pressures should be observed [11]. As previously noted in case of hemangiomas usually the enucleation (peripheral location), rarer a resection (central location, extensive tumor), is method of choice. In peripheral position of a FNH, the atypical liver resection is method of choice. Adenomas and tumors of unknown dignity should be resected for oncologic criteria (atypical resection to extended hemihepatectomy) [1, 4, 10, 11, 45]. 

### 2.5. Patient Population

This study only mentioned patients with one of the three most popular benign liver lesions, the hemangioma, the hepatocellular adenoma, and the FNH. Not for all patients of our cohort, every diagnostic possibility, named in Table 3, was exhausted. Reasons for this approach were on the one hand often patients with a positive history of cancer (non-hepatogenous origin)—almost 50%—and on the other hand a unambiguously “malignancy-suspicious” liver lesion in one method of imaging. From 2004 till 2011, 40 patients (men and women) with one of these most common benign lesions of the liver underwent surgically treatment in the clinic of Dachau, Germany. The extent of surgical treatment ranged from atypical liver resection to extended hemihepatectomy. The liver resections were performed in laparoscopic and open technique. During surgical resection for all surgical specimens, a frozen section analyses was performed. Depending on the result of this examination, the extent of liver resection was determined. In our clinic generally parenchychma sparing techniques for surgical resection are preferred. For final histological results, all surgically resected liver tumors were examined in the Institute of Pathology, Rotkreuz hospital, Munich, Germany. 

## 3. Results

### 3.1. Age and Gender Distribution

At the time of the liver resection, the age of the subjects in our series ranged from 23 to 72 years with a mean of 52.8 years. The ratio of men to females was 1.7 : 1. Tables 4 and 5 show the relevant important demographic information, age, and gender distribution divided into the three most common benign lesions of the liver, of our own patients collective compared to the literature. These tables show a higher mean age of patients in our collective and a less pronounced gender distribution. Reasons for these results are on the one hand the high number of patients with a history of cancer (non-hepatogenous origin) and on the other hand the lower number of patients in our collective compared to the literature. For instance, patients with an adenoma of the liver and a positive history of cancer would not be considered, the median age for patients with a adenoma totally was 42.1 years, and the gender distribution was 4 : 1. A similar pattern holds for the FNH and hemangioma.

### 3.2. Indication of Treatment

In patients with asymptomatic liver lesions, surgical intervention was mainly indicated since a primary or secondary malignancy could not be excluded with certainty. In case of symptomatic liver lesions, the indication for surgery was conducted on the one hand because of clinical symptoms, but on the other because a primary or secondary malignancy could not be preoperatively excluded with certainty by means of morphological imaging. Most often patients of our series complained about right upper abdominal pain. Our patients also reported about indigestion, loss of appetite, and icterus.  Table 6 compares the results of our own collective with those of the literature regarding the indication for elective surgical procedures. 81.3% of patients having health complaints had no history for carcinoma.

### 3.3. Types of Surgery

Beside the clinical and radiological diagnosis, the tumor location and size determine the extent and type of surgery.  Table 7 shows the distribution of the respective surgical procedure for benign liver lesions of our own subjects in comparison to the literature.

The distribution of the respective applied surgical procedure shows in our collective of subjects as well as the collective of the reference centers a selected group. Therefore, these results do not reflect the expected approach in the surgical treatment of benign liver lesions. Normally in the vast majority of cases, atypical resections or enucleations would be expected and less segmental resections, resections of more than one segment, or hemihepatectomies.

Table 8 presents the size of operations for the three most common and atypical benign liver tumor entities of our patient cohort. With regard to hemangioma or FNH in more than 75% of all cases, an atypical or one segment resection was conducted. Larger and especially central hemangioma and FNH should be treated with a resection of more than one segment of the liver or even with a hemihepatectomy. Depending on the tumor size and location more than 70% of adenomas were oncologically treated with a segmental resection or with a resection of more than one segment.

### 3.4. Morbidity and Mortality

The low mortality rates result from the generally good preoperative liver morphology and function in patients with benign liver lesions. The rate of postoperative complications (minor) in our collective amounts in total for more than 15%. These results emphasize the need of a strict indication for liver resection, especially in primary asymptomatic patients (Table 9). The rate of surgical revision in our cohort was 0%.

The mortality and morbidity of laparoscopic liver resections in our own collective are presented in Table 10. The results are divided into the three most common benign liver lesions and show on the one hand a shorter hospitalization of patients and on the other a lower complication rate than open surgery.

### 3.5. Patients with a History of Malignant Tumor

Table 11 shows the number of subjects in our own collective where in the context of a history of cancer (non-hepatogenous origin) a lesion of the liver was noticed. In these cases a hepatic metastasis could not be radiologically excluded with certainty. Therefore the indication for resection of the liver lesion was set wider with regard to these subjects. Aim of this approach was on the one hand to get a definitive histology of “malignancy-suspicious” lesion and if needed to deliver an additional treatment such as chemotherapy or radiotherapy without loss of time, but also on the other to reach primarily a definitive treatment. In total, form 2004 to 2011, 185 patients with a history of cancer (non-hepatogenous origin) and a suspicion of liver metastasis were surgically treated in the Clinic of Dachau, Germany. After final histological examination, 15 (8.1%) of these “malignancy-suspicious” liver lesions revealed a benign result. 

### 3.6. Compliance of Suspected Diagnosis and Operation-Histology

Table 12 presents the results of postoperative histological examination in comparison to the original diagnosis.

In 12% of cases a lesion of the liver was not known preoperatively and noticed during a laparoscopic cholecystectomy. In order to get histology, this incidental findings were completely laparoscopically, atypically resected. The subsequent histological examination showed uniformly the diagnosis of a hemangioma. In patients with a positive history of malignant disease, every intrahepatic mass of uncertain dignity was resected.Our high rate of 56% preoperative misdiagnosis for the hemangioma is therefore the result of including patients without a preoperative diagnosis and patients with limited preoperative imaging. If patients with a history of carcinoma or incidental perioperative findings are not considered in the analysis, there is an acceptable agreement of 76% of the results. With regard to FHN and adenoma, preoperative diagnosis was primarily uncertain or unreliable, this being particularly relevant forFNH with 58% and for adenoma with 72% when taking into account the patients with a history of cancer.

## 4. Discussion

The continuous development of imaging techniques enables to diagnose asymptomatic liver lesions with an increasingly high sensitivity and specificity [11, 18, 32–36, 38]. Based on the morphological and functional behavior of the various imaging procedures, the dignity of these lesions may partly be determined unambiguously [1–4, 18, 32–36, 38]. This assignment and classification is more difficult for patients with a positive malignant, non-hepatogenous history of tumor (especially in case of a colorectal and breast carcinoma). 

**Figure 5:**
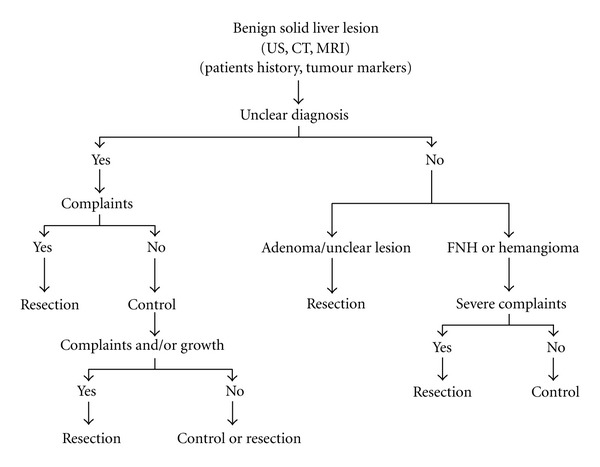
Algorithm for management of solid liver lesions (mod. from Terkivatan et al. [54]).

In patients without history of malignant tumor, the FNH and the hemangioma are relatively straightforward to diagnose [3]. In the diagnosis of a hemangioma, the CM sonography, CT, or MRI is approximately equally effective [1, 3, 11, 18, 32–35]. With an MRI, a hemangioma can be diagnosed with a sensitivity of >95% and a specificity of 95% [1, 3, 11, 18, 32–35]. Method of choice in diagnosis of FNH is the MRI with liver-specific CM (e.g., gadoxetic acid; disodium salt (Primovist)). The sensitivity is >95%; the specificity is >95% [3, 11, 18, 32–35]. The diagnosis of an adenoma is often difficult. Method of choice is the MRI with liver-specific CM (e.g., gadoxetic acid; disodium salt (Primovist)) [1, 5, 11, 18, 32–35]. The diagnosis of a hepatocellular adenoma is only in premenopausal women with noncirrhotic liver, in case of the “classical” steroid-associated solitary adenoma, quite trivial to make [5]. Sometimes a hepatocellular adenoma and a FNH cannot be clearly differentiated [5]. Gradzioli et al. exposed in their study that gadoxetic acid-enhanced MRI (Primovist) facilitates the differentiation of hepatocellular adenoma and FNH with a sensitivity of 92% and specificity of 91% [28]. In the arterial phase the gadoxetic acid contrast enhancement of FNH (mean 94.3%  ± 33.2) was significantly higher than that of adenomas (mean 59.3%  ± 28.1) (*P* < .0001) [28]. In the hepatobiliary phase, the lesion to liver contrast of hepatocellular adenomas showed strong negative values (mean −0.67 ± 0.24) and of FNH demonstrated minimally positive values (mean 0.05 ± 0.01) (*P* < .0001) [28]. Belghiti et al. [12] offered in their study with the aim of distinguishing FNH/adenoma, which in 83% of cases adenomas could be diagnosed preoperatively correctly (ultrasonography and CT). It was also apparent that all preoperative suspected diagnoses (ultrasound and CT) of FNH (18 cases) could be confirmed postoperatively, histologically (sensitivity 100%). But the histological analysis of 18 other cases also showed the diagnosis of FNH. In these patients preoperatively no unambiguous diagnosis (ultrasonography and CT) was possible (specificity 50%). Particularly problematic is the fact that postoperative, histological examination offered three HCC which were preoperative under the presumptive diagnosis of benign lesion of the liver [12]. These results emphasize the diagnostic difficulties in unclear, often asymptomatic liver lesions. Despite extensive diagnostics, the dignity of an asymptomatic liver lesion ultimately remains unclear in 10–40% of cases till surgical intervention and subsequent histology [5, 6, 11]. These points mentioned the problems in the treatment of asymptomatic liver lesions. On the one hand, an unnecessary operation with potential morbidity and mortality should be avoided; on the other often preoperatively malignancy could not be clearly excluded [5–9, 11] or a transformation into a malignancy (HCC) is feared [1, 3, 10, 23–30]. In the treatment of liver lesions, especially in case of an asymptomatic tumor, a gradual adapted approach is necessary [4, 11]. Especially with regard to questionable dignity, a critical assessment of the individual benefit-risk should be conducted [1, 4, 10, 11] (Figure 5). In our cohort despite the gradual adapted approach, often surgery was indicated by one malignancy suspect imaging. Reasons for this procedure were the high numbers of cases; the dignity of the lesion remains unclear despite extended diagnostics, to safe cost and especially the high number of patients without history for carcinoma having health complaints. In cases of asymptomatic lesions of the liver a gradual adapted approach was elected in our cohort.

Initially often close monitoring of liver lesions occurs without any major risk to the patient [4, 54]. In medical literature a correlation between tumor size and the occurrence of possible clinical symptoms is offered [11, 47]. In patients with unspecific symptoms primarily, a conservative therapy should be carried out. D'Halluin et al. showed that up to 100% of nonspecific symptoms are to relieve with conservative therapy, especially after finishing oral contraception [48]. Studies found out that only a total of about 5% of liver lesions is primarily treated surgically [1, 3, 5, 11]. 

The recommendations for surgical management of benign liver lesions are based on the results of retrospective analysis or case reports with fewer than 60 patients (medical evidence 3-4) [10]. 

At diagnosis of hepatocellular adenoma, surgical resection is indicated, because of the potential risk of malignant transformation and a possible life-threatening bleeding complication, even in case of a definite tumor regression and discontinuation of oral contraceptives [11, 55, 56] as well as absence of clinical symptoms [11, 55]. This indication should be regardless of the size of the adenoma [1, 11, 55, 56], especially in inflammatory adenomas on MRI and the possibility of beta catenin expression. In our cohort patients having a hepatocellular adenoma had health complaints in 86%. Only in 14% of cases surgery was indicated because of asymptomatic, preoperatively known adenoma.

Necrosis, hemorrhage, and thrombosis of benign lesions of the liver may often complicate the diagnosis [57, 58]. In such cases only the complete resection of the lesion can reach a definitive diagnosis.

The implementation of a percutaneous fine-needle biopsy (FNB) is discussed controversially in the literature. Due to the low expressiveness and lack of therapeutic consequences in most cases, FNB should not be performed [12, 40–42]. Only 34–40% of FNB histologies are consistent with the histology of the surgical preparation [4, 10]. In addition to a morbidity of 0.5% and a mortality of 0.05% [1, 4, 10], the FNB contains the risk of possible seeding of malignant cells through the puncture of a possible hepatocellular carcinoma (HCC). Studies have demonstrated that in case of a puncture of a liver lesion up to 2% seeding of malignant cells in the needle tract can occur [43, 44]. In accordance to these facts in our cohort, no FNB had been performed.

Asymptomatic hemangiomas do not require therapy [1, 3, 5, 10, 11]. In the current literature, there is disagreement whether asymptomatic hemangiomas depending on the size should be surgically treated. None of our patients had an acute rupture of the hemangioma, and also in the world literature, only a total of 97 cases of hemangioma rupture were published and only in 47.4% of cases (46) a spontaneous, life-threatening hemangioma rupture occurred [19]. Further investigation showed that these spontaneously ruptured hemangiomas had a mean size of 11.2 cm [19]. These studies reached the conclusion that a spontaneous rupture of a hemangioma occurs only very rarely and a prophylactic resection should only be done in case of specific requirements and especially for a size of the hemangioma >11 cm [1, 10, 19, 41]. For example, in patients with a large hemangioma (>11 cm) before a scheduled pregnancy, a definitive treatment should be discussed [10, 19]. Methods of choice in an acute hemangioma rupture are the immediate optimization of coagulation and sometimes a TAE [1, 10, 11, 18]. TAE treatment of a hemangioma is difficult and due to the aberrant collateral arterial circulation making almost improbable to stop the multiple inflow from different feeding arteries. The indication for surgical intervention should be provided in case of a high growth trend (>3 cm in 12 months), in symptomatic compression symptoms, or recurrent pain, which may correlate with hemorrhage into the lesion [18, 19]. Method of choice is the enucleation with minimal loss of parenchyma or a sparingly liver resection [10, 11, 18, 21]. In giant centrally located hemangioma a total vascular exclusion of the liver is a useful technical maneuver to save blood while dissecting it. The second key point to significantly lower the blood losses is reducing the surgically resection time [10, 18, 21]. None of our patients had an acute bleeding complication or rupture of the hemangioma. Patients of our cohort with a postoperatively ensured hemangioma had preoperatively rarely health complaints (29%), but often a moot malignancy (71%) and often a positive history for carcinoma (62%). In case of moot malignancy and history for carcinoma, extended surgical resections (liver segment resection) were performed in our clinic. Main reason for this approach was to remove a possible malignant tumor with sufficient safety distance. In case of a preoperatively know clinical symptomatic hemangioma and no history of carcinoma, always a parenchyma sparing technique was performed.

At the diagnosis of FNH, primarily no surgical intervention is indicated [1]. Large, displacing growing FNH nodes may require surgical treatment [5, 10]. This is particularly important for women wishing to have children. During pregnancy, it can lead to a progressive growth of the FNH because of hormone influences [10]. In case of an unambiguous preoperative diagnosis and indication for surgical intervention, the atypical resection is the treatment of choice [3, 5, 10, 11]. Often, the preoperative differential diagnosis of adenoma cannot be excluded with certainty. Gadoxetic acid-enhanced MRI enables the differentiation of FNH from adenoma best [28]. In these cases a surgical resection is indicated [11, 24]. In patients with a prior history of carcinoma (especially colorectal cancers and breast cancers, etc.), within a cancer followup a lesion in the liver was noticed, and hepatic metastasis could radiologically not be excluded; the indication for resection of the liver lesion should be set wider [10, 11, 24, 29, 30]. One aim of this approach is to get a definitive histology of the “malignancy-suspicious” lesion and if needed to deliver an additional treatment such as chemotherapy or radiotherapy without loss of time, but also to reach primarily a definitive therapy [1, 11, 29, 30]. Because of the high number of patients of our cohort with a positive history of cancer (47.5%) and radiologically not safe metastasis exclusion (84.2%), this approach was also chosen among these patients.

Requirements for a surgical intervention of benign lesions [4] are as follows:

- low surgical mortality (<1%),
- low morbidity,
- avoidance of blood transfusion,
- good long-term results.

Our study and the current literature revealed that these conditions are fulfilled (see Section 3) and an elective liver resection currently can be considered to be safe and effective [4, 12]. The mortality of our cohort and in the literature was 0–0.9% [11, 42, 47, 53]. In contrast, the mortality in event of an emergency surgical intervention for acute bleeding adenoma in the literature is stated 5–10% [40, 42].

The postoperative morbidity in our collective in total was 16.3%, whereas no major complications occurred and no revision surgery had to be performed. This result of the morbidity for liver resections at benign liver lesions is generally equivalent to medical literature (7–28%) [11, 42, 47, 53]. Typical postoperative complications are a right-sided serothorax, a biliary leakage, a biloma, a postoperative bleeding, a subphrenic seroma/abscess, or a liver failure [5, 59]. To avoid such complications, a careful approach should be chosen for the liver resection. As already illustrated, for the lowest possible intraoperative blood loss the liver resection can be performed, for example, with the waterjet [4, 51, 52]. For this reason liver resection with the waterjet is standard in our clinic. In addition, a thorough ligation of bile ducts should be ensued, a continuation of blood flow in the portal venous and arterial system of healthy liver parenchyma (selective liver occlusion) should be performed, intraoperative blood losses should be avoided, and a thorough sealing of resection surface should be done (e.g., local fibrin) [4, 51]. Infective complications can be reduced by perioperative administration of antibiotics [10].

In case of symptomatic benign tumors of the liver, surgical intervention in 80% of cases leads to a decrease of complaints [4, 54]. Rarely the preoperative symptoms persist and sometimes even new complaints occur because of the surgical intervention [4]. These points illustrate the strict obligation of indication for surgical intervention in case of benign liver lesions. 

Medical literature offers a low postoperative complication rate, in laparoscopic and open liver resections [50, 60]. With appropriate selection, a laparoscopic liver resection should primarily be made, because shorter hospitalization and fewer minor complications, with identical major complications are recorded [4, 10, 50]. These results were also confirmed in our collective. The rate of complications (minor and major) for liver resection in open technique was 16.5% and 5% for laparoscopic technique. Also the hospitalisation was shorter in case of laparoscopic surgery (9 days versus 11 days). For these results it is critical to note that currently extended liver resections are more frequently, like in our collective, performed in an open technique and for these interventions are to be expected both higher morbidity and a longer hospitalisation. In the medical literature studies with larger numbers of subjects are missing, where extensive liver resections for benign lesions in laparoscopic and open technique relating to mortality, morbidity, and hospitalization are compared. Smaller studies have already shown that even hemihepatectomy can be safely performed laparoscopically [10, 61]. Currently, in the literature the implementation of extensive laparoscopic and laparoscopic assisted liver resection is critically discussed [3, 4, 11, 50, 61]. The laparoscopic liver resection has disadvantages primarily in case of extended, central findings in the exact three dimensional orientation of the surgeon, such as the preparation of the great vessels. Bleeding complications are the most common reason for conversion to open liver resection [50, 61]. Other disadvantages of laparoscopic surgery are often a higher time exposure, the higher costs, and the dependence on the surgeon [4]. Nevertheless, in the future in case of a surgical treatment of a benign liver tumor, laparoscopic liver resection by experienced surgeons will be the gold standard [10].

## 5. Conclusion

Despite continuous improvement of diagnostic possibilities, the dignity for up to 40% of incidentally detected liver lesions cannot be determined reliably till the final postoperative histology. In case of uncertain diagnosis, especially in patients with positive history of a malignant tumor or the suspected diagnosis of hepatocellular adenoma, surgical resection is indicated. In case an asymptomatic hemangioma or FNH can be preoperatively detected with certainty the indication for surgery must be very reluctant. In case of symptomatic liver lesions, surgical resection should only be indicated with tumor-specific symptoms. In the remaining cases of benign lesions of the liver, a “watch and wait” strategy is recommended. Due to the continuous improvement of surgical techniques, liver resection should also be done in the laparoscopic technique in case of more than one liver-segment resection or hemihepatectomy. Laparoscopic surgery has lower morbidity and shorter hospitalization than open technique.

## Figures and Tables

**Figure 1:**
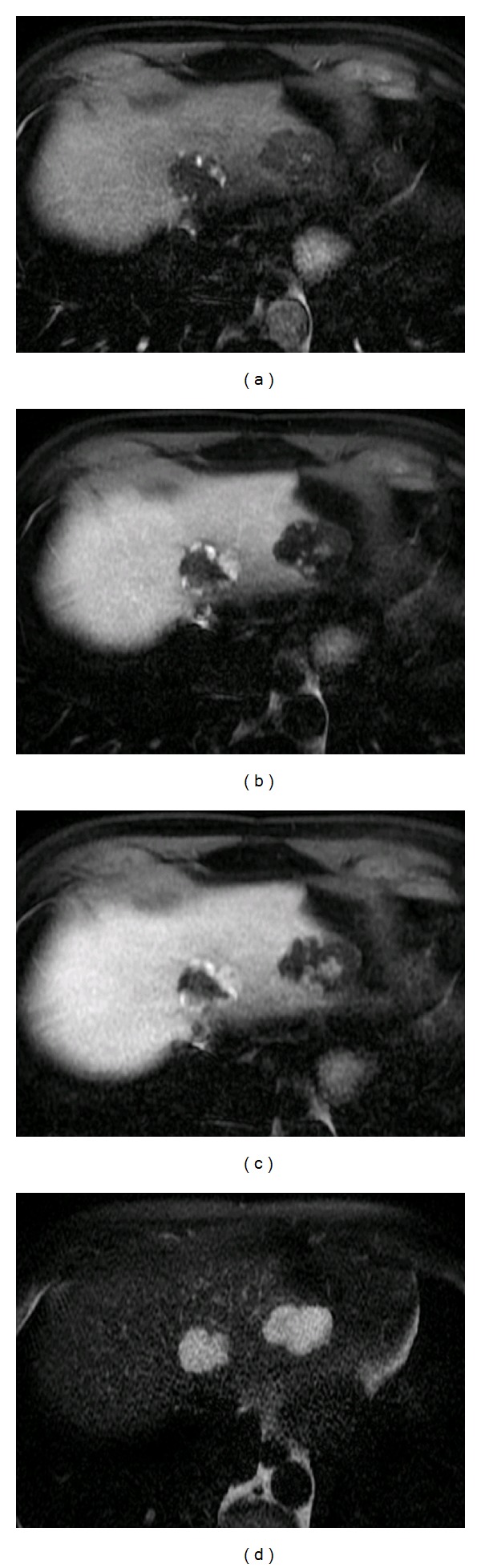
Two hemangiomas in gadolinium enhanced MRI (contrast medium: gadoxetic acid; disodium salt (Primovist, Eurokontrast GmbH, Heidelberg), scanner: GE Signa HDxt 1,5T (General Electric Company, USA)): peripheral nodular enhancement in T1 FS early arterial contrast phase (upper left): (T1 LAVA FS dynamic FA80, TR 185 TE 4,2), progressive centripetal enhancement in T1 FS late arterial (upper right): (T1 LAVA FS dynamic FA80, TR 185 TE 4,2) and portal-venous phase (lower left): (T1 LAVA FS dynamic FA80, TR 185 TE 4,2). Typical ill-shaped intermediate (less than in cysts) hyperintensity in T2 (lower right): (T2 FRFSE FS FA 90 TR 2500 TE 94,16). Lesion in left lobe is partially clotted with thrombosis and shows less enhancement.

**Figure 2:**
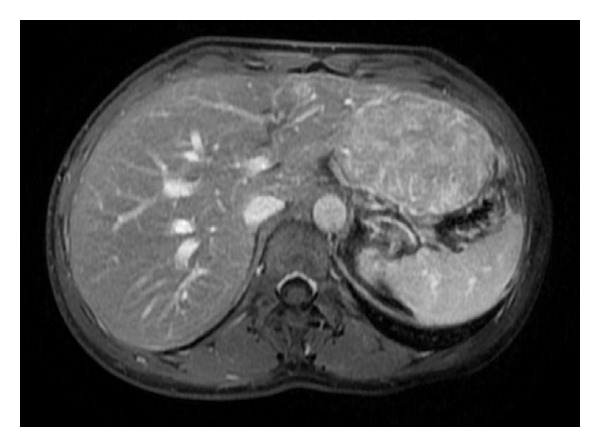
Focal nodular hyperplasia in gadolinium-enhanced MRI (contrast medium: gadoxetic acid; disodium salt (Primovist, Eurokontrast GmbH, Heidelberg), scanner: GE Signa HDxt 1,5T (General Electric Company, USA)): inhomogeneous hyperintensity on T1 FS in portal-venous contrast phase (T1 FSPGR FS FA12, TR 4,24 TE 2,04, TI 7).

**Figure 3:**
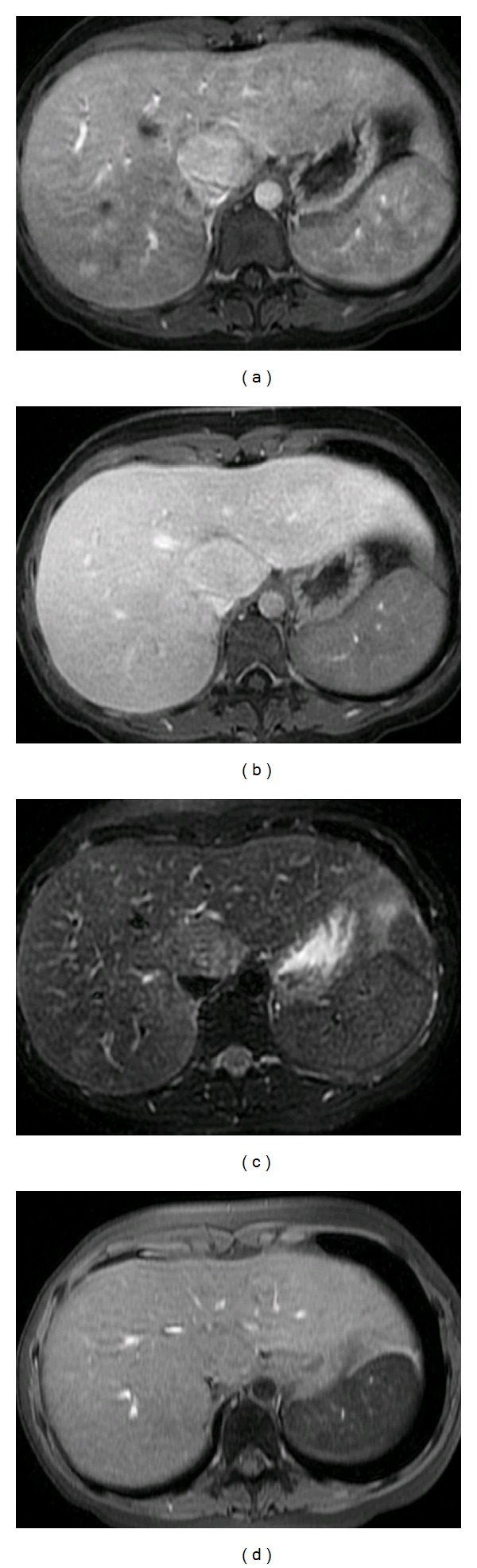
Segment 1 adenoma in gadolinium-enhanced MRI (contrast medium: gadoxetic acid; disodium salt (Primovist, Eurokontrast GmbH, Heidelberg), scanner: GE Signa HDxt 1,5T (General Electric Company, USA)): hyperintens. in T1 FS arterial contrast phase (upper left): (T1 LAVA FS dynamic FA80, TR 185 TE 4,2), partial equilibration to liver isointensity in T1 FS late portal-venous phase (upper right): (T1 FSPGR FS FA12, TR 4,24 TE 2,04, TI 7), slight hyperintensity on T2 FS (lower left): (T2 FRFSE FS FA 90 TR 2500 TE 94,16), and isointensity in unenhanced T1 fat sat. (lower right): (T1 LAVA FS dynamic FA80, TR 185 TE 4,2).

**Table 1:** Surgically relevant tumor entities [37].

	Pseudotumors^∗^	Benign neoplasia
Hepatocellular tumors	Focal nodular hyperplasia (FNH)	Hepatocellular adenoma
Endothelial tumors		Hemangioma
Biliary tumors	Von Meyenburg complex	Biliary cystadenoma
		Biliary duct adenoma
Mesenchymal tumors	Hamartoma
Connective tissue tumors	Lipoma, angiolipoma, fibroma, leiomyoma
Mixed-cellular tumors		Teratoma

*Regenerative and real-neoplastic tumors.

**Table 2:** Tumour-associated demographics [1, 3, 10, 11, 18].

	Prevalence	Age	F: M	Location	Size	Specialties
Hemangioma	5–20%	35–65	2–6 : 1	Subcapsular 90% unifocal	<5–30 cm	Synchronic hemangioma in skin, lung, or brain (10–15%); partly pregnancy-associated increase of size; with partial thrombosis often acute pain; rarely DIC (Kasabach-Merritt syndrome)

FNH	2-3%	30–50	8 : 1	Subcapsular 80% unifocal	<5–15 cm	Growing: association with OC; rarely clin. symptoms

Adenoma	Rare (incidence: 0,3: 100000 pat. pera^∗^)	25–45	10 : 1	Subcapsular often unifocal	5–15 cm rare up to 30 cm	Arise and growth: association with OC (>5 years), diabetes mellitus, androgen or steroid use, male gender, glycogen storage disease, often symptomatic

^∗^a: year, OC: oral contraceptive, DIC: disseminated intravascular coagulopathy.

**Table 3:** Morphology of the most common benign lesions in imaging techniques [1, 3, 10, 11, 18, 33–35].

	Ultrasonography	Triphasic CT	MRI	18F-FDG PET scan	CT angiography
Hemangioma (Figure 1)	More often: cavernous (high flow): heterogeneous, hypoechoic, sometimes calcifying More rare: capillary (low flow): homogeneous, hyperechoic, sharp limited, no halo Doppler: low flow, low index, absence of spectral broadening	Early phase: iridic diaphragm phenomenon with peripheral nodular enhancement Late phase: CM- enhancement rise, determination of the size	Peripheral enhancement, centripetal progression, T1: hypo intense T2: hyperintense Sensitivity >95% Specificity 95% 10% atypically	No uptake or photopenic defect compared to liver baseline	Cotton wool pooling of contrast, normal vessels without AV shunt, persistent enhancement

FNH (Figure 2)	Homogeneous, iso-, hypo- or hyperechoic, Central hyper echoic area Central aterial signal (50–70%: central scar) Doppler: high flow, spokes phenomenon, spectral broadening	Isodense with liver, Central low density Scar Arterial phase: homogeneous strongly enhance	Native: isodense T1: isodense T2: isodense hyper intense scar sensitivity >95% specificity >95%	No uptake	Hypervascular 70%; centrifugal supply

Adenoma (Figure 3)	Unspecific, Hypo- or hyper echoic Hemorrhage or necrosis: heterogeneous, anechoic center Doppler: variable flow, spectral broadening	Homogenous > heterogeneous Peripheral feeders filling in from periphery	T1 Gd: hyperintense T2: hyperintense (intralesional fat) capsule necrosis: T1: hypointense T2: hyperintense bleeding: T1 + T2: hyperintense	No uptake uptake If transformation to HCC in 30% of the cases	Hypervascular; large peripheral vessels; central scar if hemorrhage

CT: computed tomography, MRI: magnetic resonance imaging, FNH: focal-nodular hyperplasia CM: contrast medium, HCC: hepatocellular carcinoma.

**Table 4:** Mean age in years of the subjects of our own collective and the literature.

	Dachau	Zülke et al.	Charny et al.	Weimann et al.
		[11]	[47]	[42]
Hemangioma	54.9	49.5	52	47.6
FNH	54.3	36	38	35.3
Adenoma	52.3	40	34	34

**Table 5:** Gender distribution (females: men) of the subjects of our own collective and the literature.

	Dachau	Zülke et al. [11]	Charny et al. [47]	Weimann et al. [42]	Skalický et al. [5]
Hemangioma	1.6 : 1	1.2 : 1	3 : 1	2.9 : 1	2.3 : 1
FNH	2 : 1	2.5 : 1	9.5 : 1	11.5 : 1	2.1 : 1
Adenoma	1.3 : 1	2.4 : 1	5 : 1	3.9 : 1	1 : 2

**Table 6:** Indications for surgery of the three most common benign liver tumors (multiple answers in our own collective and Charny et al. [47]).

	Number of patients (*n*)			Health complaints (%)			Moot malignancy (%)		
	Dachau	Zülke et al.	Charny et al.	Dachau	Zülke et al.	Charny et al.	Dachau	Zülke et al.	Charny et al.
		[11]	[47]		[11]	[47]		[11]	[47]
Hemangioma	21	12	39	29	58	59	71	25	33
FNH	12	21	18	17	42	44	92	33	61
Adenoma	7	15	8	88	33	37	57	67	75

**Table 7:** Distribution of surgical procedures.

	Dachau	Zülke et al. [11]	Charny et al. [47]	Weimann et al. [42]
	*n* (%)	*n* (%)	*n* (%)	*n* (%)
Atypical resection	20 (46)	15 (28)	33 (48)	84 (50)
Segmental resection, resection of more than one segment	19 (44)	32 (60)	12 (18)	38 (22)
Hemihepatectomy	4 (9)	6 (11)	23 (34)	47 (28)

Total	43	53	68	169

**Table 8:** Distribution of tumor-specific surgical procedure in our collective of subjects.

	Atypical resection	Segmental resection	Resection of more than one segment	Hemihepatectomy	Total
Hemangioma	11	6	2	2	21
FNH	6	3	1	2	12
Adenoma	2	4	1	0	7
Others	1	1	1	0	3

**Table 9:** Mortality and morbidity of our subjects after resection of benign liver tumors in comparison to the literature.

	Mortality	Morbidity^∗^	Revision	Hospitalization^∗∗^
Weimann et al. [42]	0.6% (1/173)	24.9% (43/173)	—	—
Charny et al. [47]	0% (0/68)	20.6% (14/68)	—	8.5 d
Petri et al. [53]	0.9% (1/113)	27.4% (31/113)	—	—
Zülke et al. [11]	0% (0/55)	18.5% (10/55)	<5%	10 d
Dachau	0% (0/43)	16.3% (7/43)	0%	11 d

*Major and minor complications, ^∗∗^mean (laparoscopic and open resection).

**Table 10:** Mortality, morbidity, and hospitalization after resection of benign liver tumors during laparoscopic liver resections in our collective and share of laparoscopic procedures in relation to the total number of liver resections.

	Mortality	Morbidity^∗^	Hospitalization^∗∗^	Distribution^∗∗∗^
Hemangioma	0% (0/7)	14.3% (1/7)	7 d	33.3%
FNH	0% (0/8)	0% (0/8)	11 d	66.7%
Adenoma	0% (0/4)	0% (0/4)	9 d	57.1%

Total	0% (0/19)	5% (1/19)	9 d	47.5%

*Major and minor complications, ^∗∗^mean (laparoscopic resection), ^∗∗∗^percentage distribution of laparoscopic to open resections of the liver.

**Table 11:** Number of patients with a history of malignant tumor disease where hepatic resection was performed.

	Positive history for carcinoma	Radiological, no safe exclusion of a metastasis
Hemangioma	13	12
FNH	4	3
Adenoma	2	1

**Table 12:** Consistency of the preoperative diagnosis with the final postoperative histology.

	Dachau	Charny et al. [47]	Zülke et al. [11]
Hemangioma	7/16 (44)	27/39 (69)	9/12 (75)
FNH	4/12 (33)	7/18 (39)	10/21 (48)
Adenoma	3/7 (43)	6/8 (75)	5/16 (31)
Preoperatively unknown	5	—	—
